# Relationship Between Glycerolipids and Photosynthetic Components During Recovery of Thylakoid Membranes From Nitrogen Starvation-Induced Attenuation in *Synechocystis* sp. PCC 6803

**DOI:** 10.3389/fpls.2020.00432

**Published:** 2020-04-15

**Authors:** Koichi Kobayashi, Yuka Osawa, Akiko Yoshihara, Mie Shimojima, Koichiro Awai

**Affiliations:** ^1^Faculty of Liberal Arts and Sciences, Osaka Prefecture University, Sakai, Japan; ^2^Department of Biological Science, Faculty of Science, Shizuoka University, Shizuoka, Japan; ^3^Department of Biological Sciences, School of Science, Osaka Prefecture University, Sakai, Japan; ^4^School of Life Science and Technology, Tokyo Institute of Technology, Yokohama, Japan; ^5^Research Institute of Electronics, Shizuoka University, Hamamatsu, Japan

**Keywords:** cyanobacterium, nitrogen starvation, phosphatidylglycerol, photosystem, pulse amplitude modulation fluorometry (PAM), thylakoid membrane

## Abstract

Thylakoid membranes, the site of photochemical and electron transport reactions of oxygenic photosynthesis, are composed of a myriad of proteins, cofactors including pigments, and glycerolipids. In the non-diazotrophic cyanobacterium *Synechocystis* sp. PCC 6803, the size and function of thylakoid membranes are reduced under nitrogen (N) starvation but are quickly recovered after N addition to the starved cells. To understand how the functionality of thylakoid membranes is adjusted in response to N status in *Synechocystis* sp. PCC 6803, we examined changes in thylakoid components and the photosynthetic activity during the N starvation and recovery processes. In N-starved cells, phycobilisome content, photosystem II protein levels and the photosynthetic activity substantially decreased as compared with those in N-sufficient cells. Although the content of chlorophyll (Chl) *a*, total protein and total glycerolipid also decreased under the N-starved condition based on OD_730_ reflecting cell density, when based on culture volume, the Chl *a* and total protein content remained almost constant and total glycerolipid content even increased during N starvation, suggesting that cellular levels of these components decrease under the N-starved condition mainly through dilution due to cell growth. With N addition, the photosynthetic activity quickly recovered, followed by full restoration of photosynthetic pigment and protein levels. The content of phosphatidylglycerol (PG), an essential lipid constituent of both photosystems, increased faster than that of Chl *a*, whereas the content of glycolipids, the main constituents of the thylakoid lipid bilayer, gradually recovered after N addition. The data indicate differential regulation of PG and glycolipids during the construction of the photosynthetic machinery and regeneration of thylakoid membranes. Of note, addition of PG to the growth medium slightly accelerated the Chl *a* accumulation in wild-type cells during the recovery process. Because PG is required for the biosynthesis of Chl *a* and the formation of functional photosystem complexes, rapid PG biosynthesis in response to N acquisition may be required for the rapid formation of the photosynthetic machinery during thylakoid regeneration.

## Introduction

A typical cyanobacterial cell contains the thylakoid membrane in addition to the plasma membrane and the outer membrane. In the thylakoid membrane, photosystem II (PSII), cytochrome *b*_6_*f* and photosystem I (PSI) are embedded in a lipid bilayer with ATP synthase and form photosynthetic electron transport chain, which, by harnessing light energy, creates an electrochemical proton gradient for ATP synthesis and a strong reductant capable of reducing NADP^+^ (Lea-Smith et al., 2016; Liu, 2016). On the surface of the thylakoid membrane, phycobilisomes (PBSs) associate with PSII and PSI as the light-harvesting antenna and transfer absorbed light energy to chlorophyll (Chl) *a* in the photosystem complexes.

Proteins are the most abundant component of the thylakoid membrane and account for 50–70% of total thylakoid components by weight (Murata et al., 1981; Chapman et al., 1983; Omata and Murata, 1983). Nitrogen (N) starvation strongly affects protein homeostasis and thus thylakoid functions including the photosynthetic electron transport (Forchhammer and Schwarz, 2019). The most prominent alteration in proteome with N starvation is extensive degradation of PBSs, which are composed of phycobiliproteins with covalently bound phycobilins and non-pigmented linker proteins. The degradation of PBSs leads to chlorosis of the N-starved cell in combination with decreased Chl *a* content. Proteins in PBSs constitute up to 50% of total protein in the cyanobacterial cell under optimal growth conditions and thus can provide massive nutrients with degradation in response to N starvation (Collier and Grossman, 1992; Görl et al., 1998). Another objective of the active degradation of PBSs may be avoidance of photodamage caused by over reduction of photosynthetic electron carriers due to the low metabolic activity during N starvation (Forchhammer and Schwarz, 2019). The levels of photosystem proteins also decrease under N-starved conditions, with PSII subunits more strongly degraded than PSI subunits (Spät et al., 2018). Thylakoid membranes become almost absent after long-term N starvation, and instead, granules of glycogen and other storage compounds accumulate in the cytosol. However, in *Synechocystis* sp. PCC 6803 (hereafter *Synechocystis*), addition of nitrate to N-starved cells induces gradual re-formation of thylakoid membranes (Klotz et al., 2016). Chlorotic *Synechocystis* cells re-green and almost completely re-establish thylakoid membranes after 48 h of recovery from N starvation.

In addition to proteins and photosynthetic pigments, glycerolipids are major and essential constituents of the thylakoid membrane. The lipid composition of the thylakoid membrane is highly conserved among cyanobacteria and chloroplasts of algae and plants, with four major glycerolipids, monogalacto-syldiacylglycerol (MGDG), digalactosyldiacylglycerol (DGDG), sulfoquinovosyldiacylglycerol (SQDG), and phosphatidylglycerol (PG) (Janero and Barrnett, 1981; Murata et al., 1981; Dorne et al., 1990). MGDG, which contains one galactose residue bound to diacylglycerol, is the most abundant lipid class in the thylakoid membrane, followed by DGDG formed from MGDG. Together with SQDG containing a sulfoquinovose in the polar head group, these glycolipids account for ∼90 mol% of total thylakoid lipids. The rest ∼10 mol% is composed of PG, the only phospholipid in cyanobacteria. These glycerolipids form a lipid bilayer of the thylakoid membrane, which avoids free diffusion of ions and allows for generating an electrochemical potential difference across the membrane for ATP synthesis. Besides providing a matrix embedded with protein–pigment complexes and ATP synthase, glycerolipids in thylakoids play essential roles in photosynthesis as structural and functional components of PSII, cytochrome *b*_6_*f* and PSI (Kobayashi et al., 2016). Thus, biogenesis of the thylakoid membrane with the functional photosynthetic machinery needs coordinated synthesis and assembly of proteins, cofactors including Chls, and glycerolipids, as represented by essential roles of galactolipids in the thylakoid membrane organization during chloroplast development in *Arabidopsis thaliana* (Fujii et al., 2019b).

In *Synechocystis*, the size and function of thylakoid membranes are reduced under N starvation but are rapidly recovered after N addition (Klotz et al., 2016). However, during this process, how the amounts of glycerolipids and other thylakoid components are organized with the thylakoid functionality remains unknown. To understand the relationship between the membrane lipid metabolism and other thylakoid-associated processes during N starvation and recovery in *Synechocystis*, we investigated changes in the content of membrane lipids and other thylakoid components and the photosynthetic activity before and after N addition to the N-starved cells.

## Materials and Methods

### Growth Conditions

*Synechocystis* cells were cultured in 1,000 ml Erlenmeyer flasks containing 500 ml of the BG11 medium (Stanier et al., 1971) for photoautotrophic growth at 30°C under continuous light (15 μmol photons m^–2^ s^–1^) with rotary shaking at 100 rpm. For the N-starved growth, cells were first grown in BG11 medium for 7 days and collected by centrifugation (1,840 × *g*, 10 min). After a wash of the cell precipitates with N-free medium (BG11_0_: BG11 without nitrate), cells were resuspended in BG11_0_ to set OD_730_ = 0.4. For the recovery growth from N starvation, cells grown in BG11_0_ for 7 days were harvested (1,840 × *g*, 10 min) and resuspended in the BG11 medium to set OD_730_ = 0.6. The cell density was measured using a spectrophotometer (UV-2600, Shimadzu, Japan or V730BIO, JASCO, Japan). For PG supplementation, 40 mM dioleoyl-PG (Sigma-Aldrich) dissolved in 100% ethanol was added to growth media at a final concentration of 20 μM PG and 0.05% (v/v) ethanol. As the negative control of PG supplementation, 100% ethanol was added to growth media at a final concentration of 0.05% (v/v).

### Determination of Absorption Spectra and Content of Chl *a* and Phycocyanin

For measurement of absorbance spectra of intact cells, an integrating sphere (ISR-2600, Shimadzu) was attached to the UV-2600 spectrophotometer (Shimadzu, Japan). Chl *a* and phycocyanin content was estimated from the absorbance of intact cells at 620 nm and 678 nm as described (Arnon et al., 1974). For accurate determination of Chl *a* content, pigments were extracted from cells with 100% methanol. Concentration of Chl *a* in the methanol extracts was calculated from the absorbance at 665 nm (Grimme and Boardman, 1972) or from the fluorescence emission at 670 nm under 435 nm excitation measured by a spectrofluorometer (RT-5300PC, Shimadzu, Japan). The Chl *a* standard sample of known concentration was used for the fluorometric determination of the Chl *a* content.

### Determination of Oxygen Evolution Rates and PSI Activity

The oxygen consumption rate and evolution rate were measured in cell suspensions at OD_730_ of 0.6 with a Clark-type oxygen electrode (Hansatech Instruments Ltd., United Kingdom) and a LED lamp (CCS Inc., Japan). Oxygen consumption was measured in the absence of light and net oxygen evolution was measured with illumination of growth light (48 μmol photons m^–2^ s^–1^). The data were normalized with the OD_730_ value. To determine the maximal photosynthetic activity, the oxygen evolution rate was measured under saturating light (1995 μmol photons m^–2^ s^–1^) and the oxygen consumption rate in the dark was subtracted from the data. The gross oxygen evolution rates were normalized with Chl *a* content of each cell suspension sample. The oxidation kinetics of the PSI reaction center Chl (P700) was measured by a Dual-PAM system (Heinz Walz GmbH, Germany) in cell suspensions at OD_730_ of 0.5. The quantum yields of the PSI photochemistry [Y(I)], non-photochemical energy dissipation due to the acceptor-side limitation [Y(NA)], and that due to the donor-side limitation [Y(ND)] were measured after 2-min illumination of either low (34 μmol photons m^–2^ s^–1^) or high actinic light condition (212 μmol photons m^–2^ s^–1^). The maximum level of P700 (Pm) was measured with saturating light irradiation before the actinic light treatment.

### Protein Extraction, SDS-PAGE, and Western Blot Analysis

For protein extraction, 20 ml of culture (OD_730_ = 0.5) was centrifuged at 1,840 × *g*, and the precipitated cells were frozen with liquid nitrogen. These cells were lysed six times by a homogenizer (Micro Smash MS-100R, TOMY, Japan) at 4,500 rpm for 20 s at 2°C. Then the powdered cells were suspended in 100 μl of the resuspend solution [1 mM phenylmethylsulfonyl fluoride and 5 mM 6-aminocaproic acid] (Barthel et al., 2013). For protein content analysis, aliquots of the suspended cells were mixed with the same volume of detergent solution [60 mM Tris-HCl (pH6.8), 2% SDS (w/v)]. A BCA Protein Assay Kit (Thermo Fisher Scientific, United States) was used with bovine serum albumin as a standard. For SDS-PAGE and Western blot analysis, 70 μl of the resuspended cell solution was mixed with the same volume of loading buffer [0.5 M Tris-HCl (pH6.8), 60% (w/v) glycerol, 10% (w/v) SDS, 350 mM dithiothreitol, 1 mg/ml bromophenol blue] and incubated at 37°C for 2 h to solubilize proteins with avoiding aggregation of membrane proteins. Then 10 μl of the resulted protein solution was loaded on polyacrylamide gels containing 10% (w/v) acrylamide to perform Laemmli SDS-PAGE (Laemmli, 1970). For visualization of protein bands, the gels were stained with Coomassie Brilliant Blue R-250 (CBB). Pre-stained Protein Markers (Broad Range) for SDS-PAGE (Nacalai Tesque, Japan) were used to calibrate the gels.

For immunodetection, rabbit antibodies against PsaA (AS06172), PsaB (AS10695), PsbC (AS111787, CP43), and RbcL (AS03037) (Agrisera, Sweden) were used at a 1:3,000 dilution. A polyclonal antibody against spinach PsbA/D (a gift from Dr. Masahiko Ikeuchi at The University of Tokyo) was used at the same dilution. These antibodies were detected with an anti-rabbit horseradish peroxidase-coupled antibody (ab97051, Abcam plc, United Kingdom) at a dilution of 1:10,000 followed by development with Western Lightning Plus-ECL (Perkin Elmer, United States).

### Lipid Analysis

Lipids were extracted as described (Bligh and Dyer, 1959) and separated by TLC. For membrane lipid analysis, a solvent system of chloroform: methanol: 25% ammonium (65: 35: 5, v/v) (Sato and Murata, 1988) was used for separation. For quantitative analysis of fatty acids attached to lipids, each separated spot was scraped off and fatty acid methyl esters (FAMEs) were prepared. FAMEs were then analyzed by gas chromatography equipped with a flame ionization detector as described (Awai et al., 2014). From the FAME data, fatty acid composition and the amount of each lipid class were calculated by using pentadecanoic acid as internal standard.

### Transmission Electron Microscopy

A 30 ml culture of *Synechocystis* cells (OD_730_ = 0.5) was harvested and fixed in 2% (w/v) glutaraldehyde/BG11 medium for 30 min at room temperature. Cells were centrifuged (1,840 × *g*, 20 min) and resuspended in the fixing buffer [2.5% (w/v) glutaraldehyde, 0.1 M sodium phosphate (pH7.2)] for 20 min at room temperature. Then the cells were harvested again (1,840 × *g*, 20 min), resuspended in the fixing buffer for 100 min at room temperature, and then stored at 4°C. Samples were then washed five times in the fixing buffer for 5 min each at room temperature, and post-fixed with 1% (w/v) osmium tetroxide in 0.1 M sodium phosphate buffer (pH7.2) for 2 h at room temperature. The fixed samples were dehydrated in a graded ethanol series and embedded in epoxy resin mixture (Epon812 mixture: TAAB, EM Japan, Japan). Ultrathin 70 nm sections were cut on a diamond knife with an ultramicrotome (Leica EM UC7, Leica, Germany) and placed on copper grids. The sections were first stained with EM stainer (Nissin EM, Japan) for 60 min and then with lead citrate for 9 min. Samples were observed under a transmission electron microscope (JEM-1400Plus, JEOL, Japan) with accelerating voltage at 100 kV.

Quantification of total thylakoid length and total area of high electron-dense inclusions in a cross-section of a *Synechocystis* cell was carried out on the transmission micrographs with ImageJ software. Small spots with the area <1,000 nm^2^ were eliminated from the analysis of the high electron-dense inclusions.

## Results

### Rapid Changes in Pigment Content in Response to N Conditions

**FIGURE 1:**
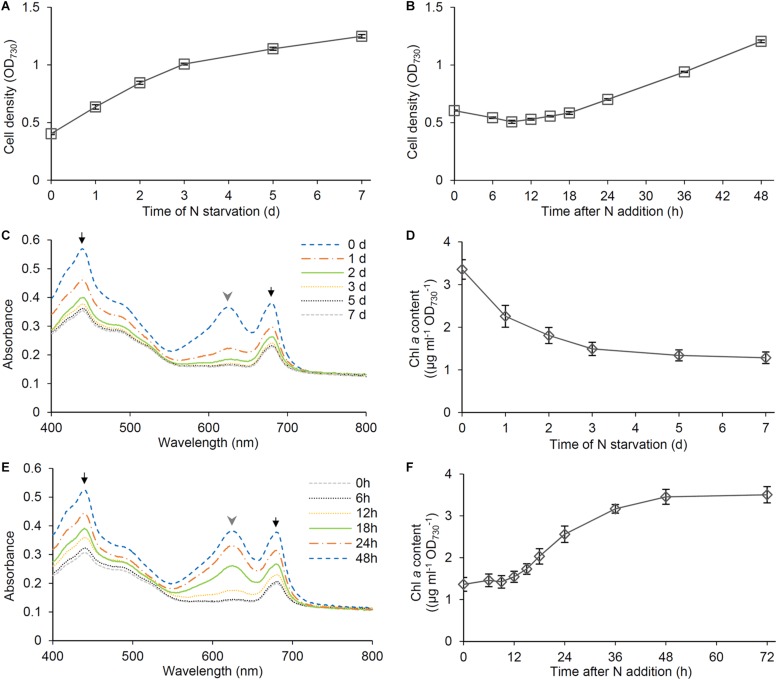
Cell growth and pigment content during N starvation and the recovery after N addition. *Synechocystis* cells were grown in the N-free medium for 7 days and then were transferred to the N-containing medium and grown for 48 h. **(A,B)** Changes of OD_730_ values of the cell culture during **(A)** N starvation and **(B)** recovery. Data are means ± SE from three independent experiments. **(C)** Absorption spectra and **(D)** chlorophyll (Chl) *a* content of the cells grown under the N-starved condition for indicated times. **(E)** Absorption spectra and **(F)** Chl *a* content of the cells during recovery from N starvation. Data are means from two independent experiments for **(C,E)** and means ± SE from six independent experiments for **(D,F)**. Black arrows and gray arrowheads in **(C,E)** indicate absorption mainly by Chl *a* and phycobilisomes, respectively.

The availability of nitrogen strongly affects growth of non-diazotrophic cyanobacteria including *Synechocystis*. In *Synechocystis*, cell density represented by OD_730_ only slowly increased under the N-starved condition (Figure 1A), which agreed with a similar increase in cell number of *Synechocystis* during N starvation (Krasikov et al., 2012). After the growth in the absence of N source for 7 days, *Synechocystis* cells were transferred to the fresh BG11 medium containing nitrate as N source. The OD_730_ values slightly decreased at the early stage of recovery but began to increase after 18 h of N addition (Figure 1B). These data are consistent with the previous report in *Synechocystis* by Klotz et al. (2016), in which cells were starved for N for the longer term (>1 month). Then we analyzed changes of pigment content based on OD_730_ during N starvation and recovery. Previous studies reported that PBSs are subject to rapid degradation in response to N starvation (Collier and Grossman, 1992; Görl et al., 1998; Deschoenmaeker et al., 2014; Spät et al., 2018). In fact, our spectroscopic analysis of intact cells using an integrating sphere demonstrated that N starvation induced a rapid decrease in the absorbance around 626 nm mainly by PBSs (Figure 1C). The absorbance around 680 and 440 nm mainly by Chl *a* also decreased during N starvation, but to a lesser extent than that by PBSs, consistent with the gradual decrease of Chl *a* content during N starvation determined by the methanol extraction method (Figure 1D). Spectroscopic estimation of phycocyanin and Chl *a* content in intact cells further supported the rapid and moderate decrease in phycocyanin and Chl *a* content, respectively, at the early stage of N starvation (Supplementary Figure S1A). Phycocyanin began to accumulate from 6 h after transfer to the N-sufficient condition and the content rapidly increased between 12 and 24 h (Figure 1E). Chl *a* showed an accumulation pattern similar to phycocyanin, but with a lower increase rate than phycocyanin (Figure 1F and Supplementary Figure S1B).

### Changes in Protein Content During the Recovery Process From N Starvation

**FIGURE 2:**
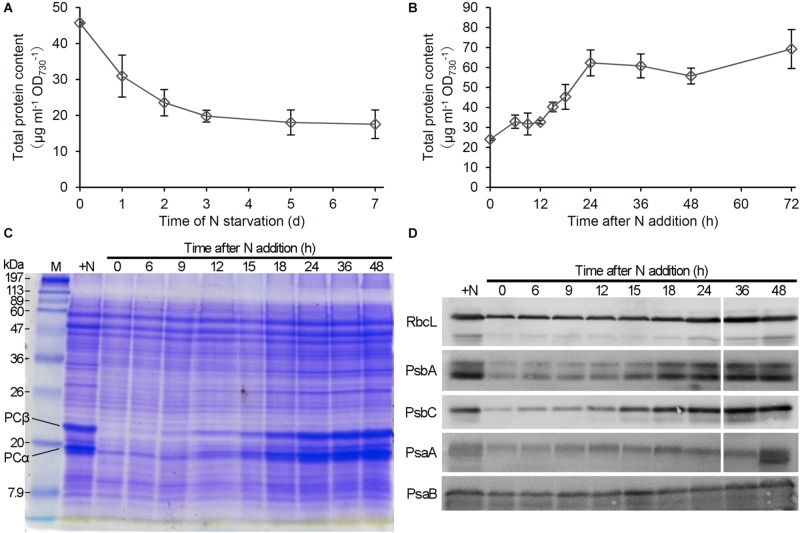
Changes in protein levels during N starvation and recovery. **(A,B)** Changes of total protein content during **(A)** N starvation and **(B)** recovery. Data are means ± SE from three independent experiments. **(C)** Gel electrophoretic patterns of total protein from *Synechocystis* cells grown in the N-containing medium for indicated times after N starvation for 7 days. PCα and PCβ indicate the bands corresponding to phycocyanin α and phycocyanin β, respectively. + N indicates proteins from cells grown under the continuous N-sufficient condition. M; protein size markers. **(D)** Immunoblot analysis of reaction center proteins of PSII (PsbA and PsbC) and PSI (PsaA and PsaB) and the RuBisCO large subunit (RbcL) in recovering cells from N starvation.

To compare the changes of protein content with those of PBSs and Chl *a* in response to N status, we performed a time-course analysis of total protein content during N starvation and recovery. As did the Chl *a* content, the total protein content based on OD_730_ gradually decreased during N starvation (Figure 2A). The protein content increased during first 6 h after N addition to the starved cells, followed by a stationary phase between 6 and 12 h and then a rapid increase until 24 h (Figure 2B). SDS-PAGE analysis with CBB staining, in which loaded protein content was normalized to OD_730_, revealed that the amounts of phycocyanin α (17.5 kDa) and phycocyanin β (21 kDa) substantially decreased with N starvation but rapidly increased between 6 and 24 h after transfer to the N-sufficient condition (Figure 2C), consistent with the spectroscopic data from intact cells. We also performed immunoblot analysis of photosynthesis-associated proteins during the recovery from N starvation (Figure 2D). The levels of reaction center proteins of PSII, PsbA, and PsbC (also called D1 and CP43, respectively), strongly decreased by N starvation, but with a small portion of them remained. By contrast, the levels of PSI reaction center proteins, PsaA and PsaB, only moderately changed in response to N starvation and recovery. The Rubisco large subunit (RbcL) also slightly decreased with N starvation and gradually increased during the recovery process, contrasting with the substantial loss of PBSs in N-starved cells and their dynamic accumulation during recovery from N starvation.

### Oxygen Metabolism During the Recovery Process From N Starvation

**FIGURE 3:**
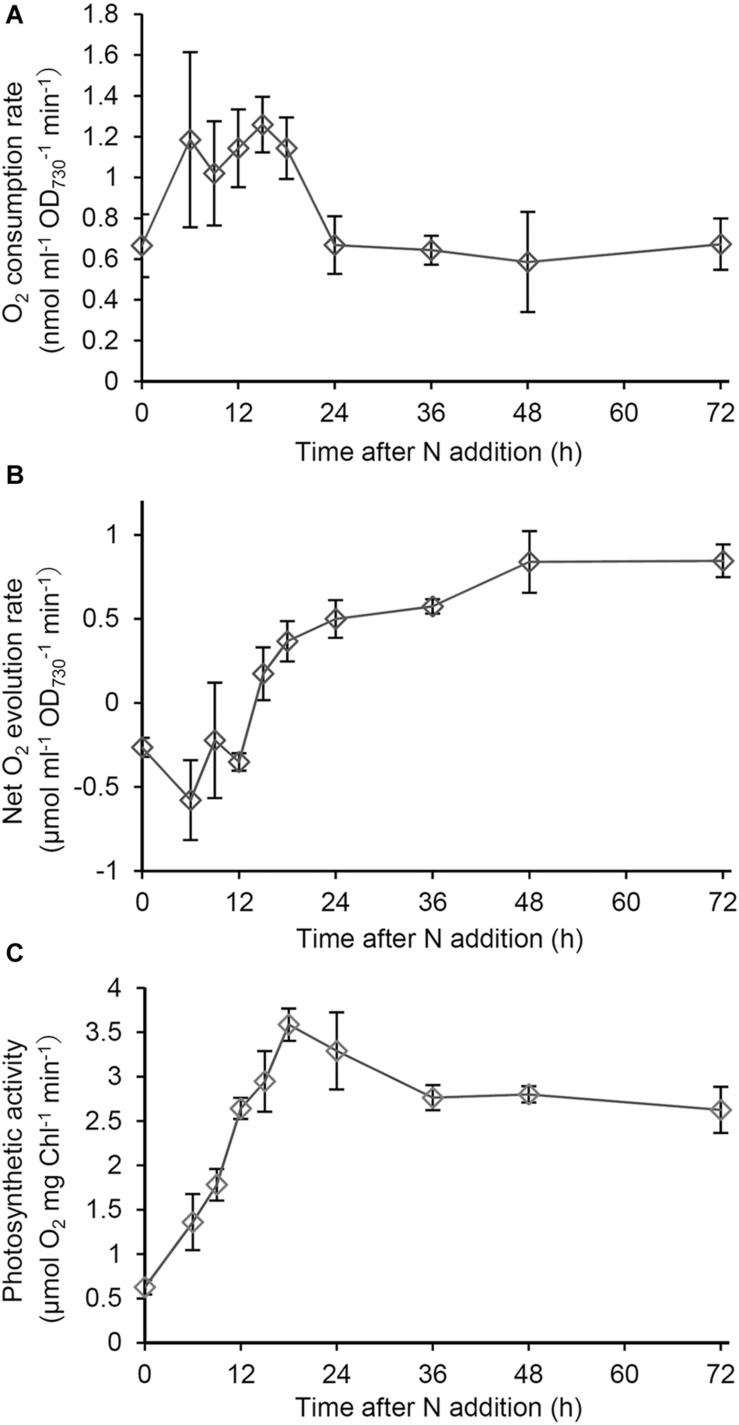
Changes in respiratory and photosynthetic activities during recovery from N starvation. **(A)** O_2_ consumption rate based on OD_730_ in the dark in *Synechocystis* cells grown in the N-containing medium for indicated times after N starvation for 7 days. **(B)** Net O_2_ evolution rate based on OD_730_ under growth light (48 μmol photon m^–2^ s^–1^) during the recovery process from N starvation. **(C)** Photosynthetic activity based on Chl *a* content, which was measured as gross O_2_ evolution rate under saturating light (1995 μmol photon m^–2^ s^–1^). Data are means ± SE from three independent experiments.

To examine the energy metabolism during recovery from N starvation, we analyzed O_2_ consumption and evolution rates in intact cells. First, O_2_ consumption by respiration was examined in the dark (Figure 3A). The O_2_ consumption rate in N-starved cells increased 1.8 times at 6 h after N addition. The high O_2_ consumption continued until 18 h after N addition, and then rapidly decreased. After the transient increase, the O_2_ consumption rate was maintained at lower levels similar to that in the N-starved cells.

Next we examined net O_2_ evolution under the growth light condition during the recovery process (Figure 3B). Until 15 h after N addition, net O_2_ evolution levels were <0, so the respiratory O_2_ consumption was higher than the photosynthetic O_2_ evolution. After 15 h, the net O_2_ evolution rate was maintained at levels above the compensation point. To examine the functionality of the photosynthetic machinery during the recovery process, we measured gross O_2_ evolution rates on a Chl *a* basis under saturating light (1995 μmol photons m^–2^ s^–1^), in which O_2_ consumption rates in the dark was subtracted (Figure 3C). The photosynthetic activity measured as the gross O_2_ evolution rate was at the low level in N-starved cells and rapidly increased in response to N addition, with peaking at 18 h after N addition and maintaining high levels afterward.

### Changes in Photosynthetic Activity During the Recovery Process From N Starvation

**FIGURE 4:**
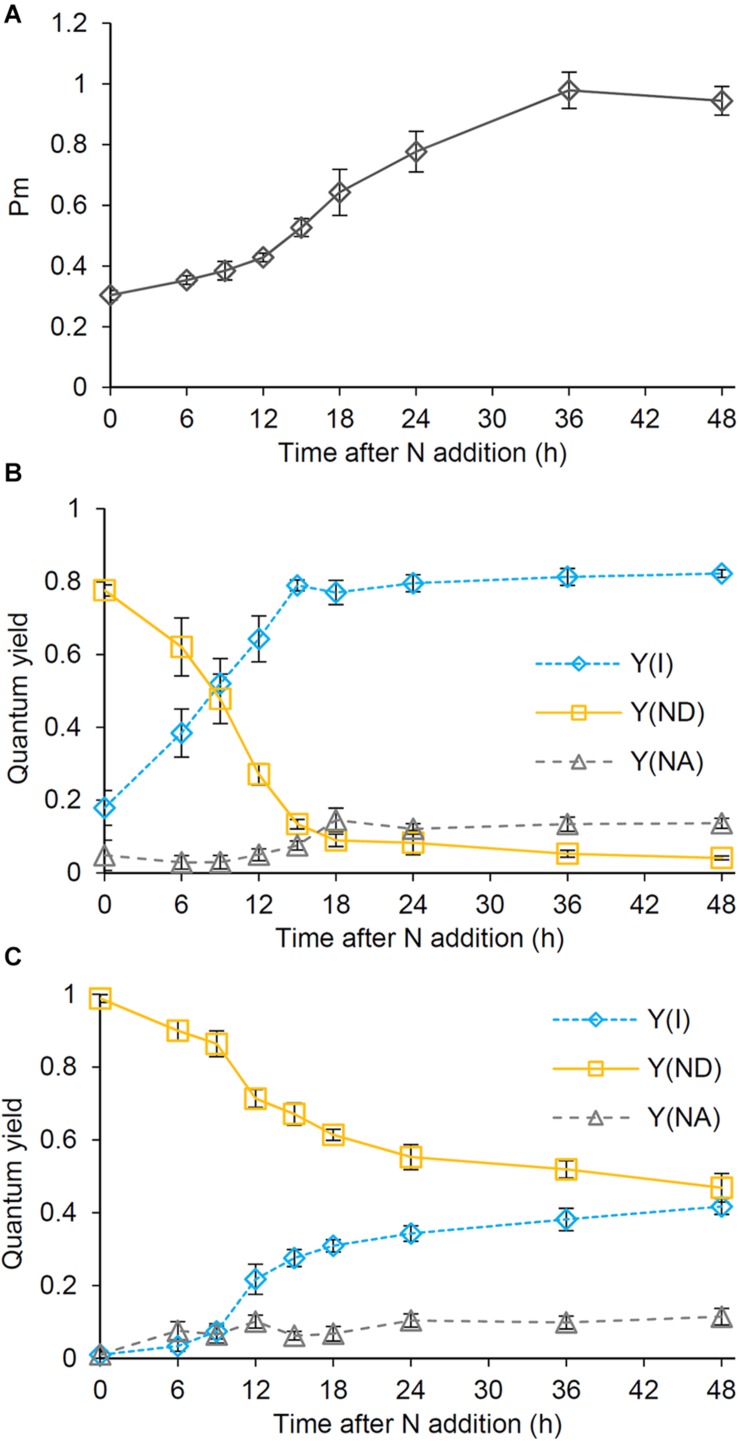
Changes in the photosynthetic quantum yield of PSI during the recovery from N starvation. **(A)** The maximum level (Pm) of the oxidized PSI reaction center chlorophyll. **(B,C)** Photochemical [Y(I)] and non-photochemical quantum yields [Y(ND) and Y(NA)] of PSI under **(B)** low (34 μmol photon m^–2^ s^–1^) and **(C)** high actinic light (212 μmol photon m^–2^ s^–1^). Y(ND) and Y(NA) reflect limitations of PSI photochemistry at the donor side and the acceptor side, respectively. *Synechocystis* cells were grown under the N-starved condition for 7 days and then grown in the N-containing medium for indicated times. Data are means ± SE from six independent experiments.

To analyze the photosynthetic electron transport activity during recovery from N starvation, we examined the oxidation kinetics of P700. The Pm levels, which suggest the size of functional PSI reaction centers, gradually increased until 36 h after N addition, with a particular increase between 12 and 18 h (Figure 4A). In N-starved cells, Y(I) representing the quantum yield of the PSI photochemistry was substantially low under the low actinic light (34 μmol photons m^–2^ s^–1^), whereas Y(ND) reflecting the donor-side limitation of PSI was high (Figure 4B). After N addition, Y(I) rapidly increased until 15 h, with Y(ND) inversely decreased. Y(NA) reflecting the acceptor-side limitation of PSI was maintained at the low level during the early recovery stage and then slightly increased at later stages. The contribution of Y(ND) was further enhanced under higher actinic light intensity (212 μmol photons m^–2^ s^–1^) (Figure 4C). By contrast, under high actinic light, Y(I) was undetectable in N-starved cells and was maintained at low levels even after N addition for 9 h. However, Y(I) rapidly increased between 9 and 12 h and then gradually moved closer to a stationary level, with Y(ND) inversely decreased. Even under high actinic light, Y(NA) was maintained at lower levels.

### Changes in Lipid and Fatty Acid Compositions During the Recovery Process From N Starvation

**FIGURE 5:**
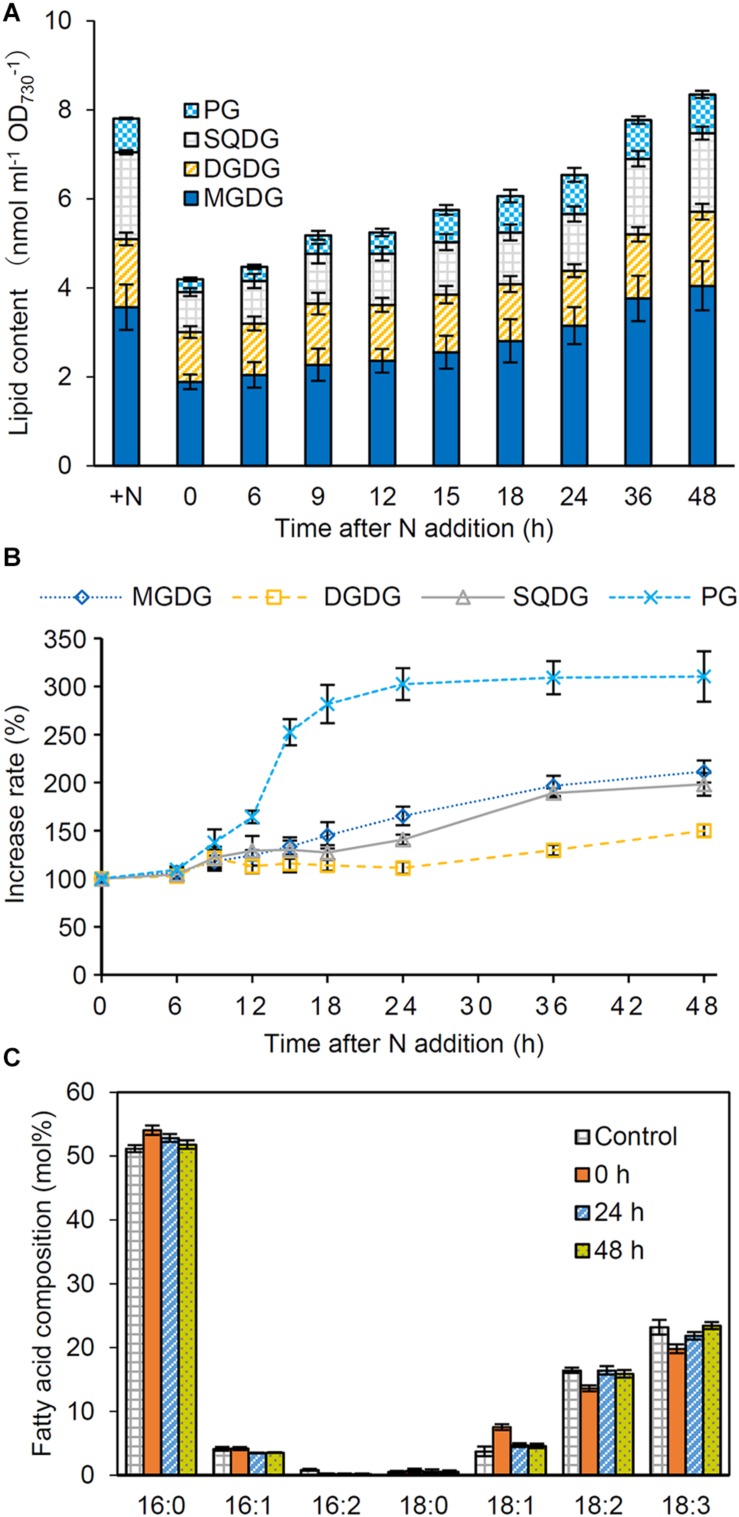
Changes in lipid content during the recovery from N starvation. **(A)** Total and individual lipid content, **(B)** the increase rate of each lipid, and **(C)** the fatty acid composition of total glycerolipids after N addition. *Synechocystis* cells were grown under the N-starved condition for 7 days and then grown in the N-containing medium for indicated times. Data are means ± SE from four independent experiments. In **(B)**, increase rates of the each lipid content relative to the initial level before N addition (0 h) were shown as percentage.

To assess whether the abundance of glycerolipids was changed in response to N conditions, we performed a time-course analysis of glycerolipid content during recovery from N starvation. In N-starved cells, total glycerolipid content based on OD_730_ decreased by half as compared with that in N-sufficient control (Figure 5A). The content gradually increased during the recovery process and reached to a steady-state level at 36 or 48 h after N addition. Of the four glycerolipids, PG showed the strongest increase in response to N addition, with its increasing kinetics being similar to or slightly faster than that of PBSs, Chl *a* and total proteins (Figure 5B). Other three lipids showed more gradual increases during the recovery process.

We also analyzed fatty acid compositions of glycerolipids in N-starved and recovered cells. In total glycerolipids from N-starved cells, proportions of 16:0 and 18:1 slightly increased and those of 18:2 and 18:3 decreased compared with those from N-sufficient control cells (Figure 5C). However, the fatty acid composition altered under N starvation reverted to the control level during the recovery process from N starvation. Changes in the fatty acid composition during the recovery process differed among glycerolipid classes; MGDG, SQDG, and PG showed transient modifications of their fatty acid compositions between 6 and 24 h after N addition with distinct patterns specific to each lipid class. Meanwhile, the fatty acid composition of DGDG was constant during recovery from N starvation (Supplementary Figure S2).

### Changes in the Amounts of Thylakoid Components in Cell Culture

**FIGURE 6:**
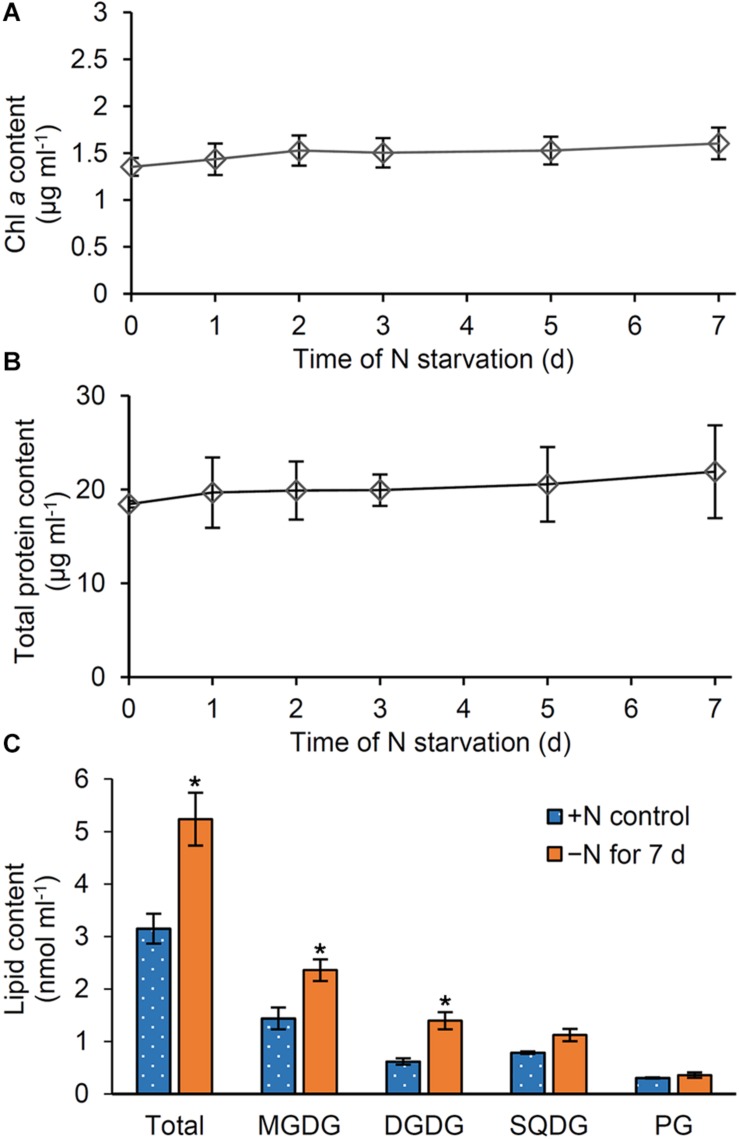
Changes in pigment, protein and lipid content based on culture volume during N starvation. Content of **(A)** Chl *a*, **(B)** total proteins, and **(C)** glycerolipids per milliliter of culture of *Synechocystis* cells. Data in Figure 1D for Chl *a*, Figure 2A for total proteins, and Figure 5A for glycerolipids were recalculated with the OD_730_ data in Figure 1A. In **(C)**, lipid content in cells grown under the continuous N-sufficient (+ N) condition or the N-starved (−N) condition for 7 days was shown. Asterisks indicate significant differences from the + N control (*P* < 0.05, Welch’s *t*-test).

Our data showed that pigments (Figure 1), proteins (Figure 2), and glycerolipids (Figure 5) were all decreased in N-starved *Synechocystis* cells based on OD_730_. To assess whether these components are actively degraded in response to N starvation or merely diluted due to cell growth, we calculated the amounts of these components based on culture volume. Whereas the amount of phycocyanin per milliliter of culture substantially decreased during N starvation (Supplementary Figure S3), those of Chl *a* (Figure 6A) and total protein (Figure 6B) were almost constant and total lipid content (Figure 6C) even increased. Of four lipid classes in *Synechocystis*, MGDG, and DGDG particularly showed increased content during N starvation based on culture volume, whereas PG content per culture volume was unchanged after N starvation for 7 days.

### Changes in Thylakoid Membrane Morphology During the Recovery Process From N Starvation

**FIGURE 7:**
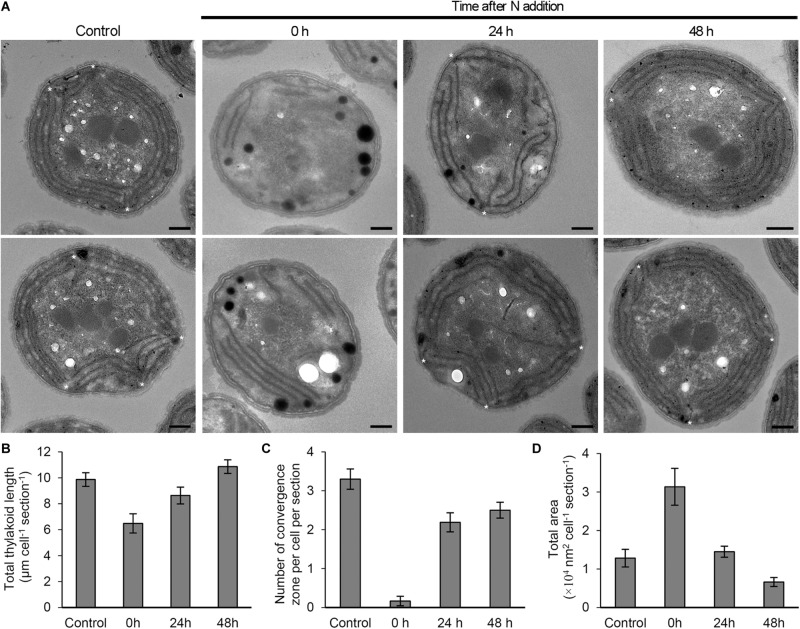
Changes in the morphology of *Synechocystis* cells in response to N status. **(A)** Ultrastructure of *Synechocystis* cells grown in the N-containing medium for indicated times after 7 days N starvation was compared with N-sufficient control cells (Control) that were continuously grown in the N-containing medium. Two images of cell ultrastructure were shown for each condition. Asterisks indicate thylakoid convergence zones. Bars = 500 nm. **(B)** Total thylakoid length, **(C)** number of convergence zone and **(D)** total area of high electron-dense inclusions in a cross-section of a *Synechocystis* cell were quantified from the transmission electron micrographs for each condition. Data are means ± SE (*n* = 10 for Control, 11 for 0 h, 12 for 24 h, and 15 for 48 h in B; *n* = 10 for Control, 18 for 0 h, 16 for 24 h, and 16 for 48 h in C; *n* = 10 for Control, 16 for 0 h, 29 for 24 h, and 27 for 48 h in **D**).

To examine the effect of N status on the thylakoid morphology, we compared the ultrastructure of *Synechocystis* cells grown under different N conditions (Figure 7). The cells grown under the continuous N-sufficient condition developed thylakoids as parallel stacks of two to four membranes along the contour of the cell membrane. In addition, some parallel sheets of thylakoid membranes formed convergence zones in close contact with the plasma membrane (Rast et al., 2019). By contrast, cells grown under the N-starved condition for 7 days had fewer thylakoid membranes and rarely formed convergence zones. In addition, N-starved cells accumulated many high electron-dense inclusions presumably lipid inclusions, cyanophycin granules, and/or polyphosphate granules. These observations were confirmed by the quantification of electron micrographs (Figures 7B–D). At 24 h after N addition, thylakoid morphology was heterogeneous: some cells showed largely or partially recovered thylakoid membranes and some severely lacked the membrane network. Two or three convergence zones were observed in cells with recovered thylakoid membranes. Many cells also contained high electron-dense inclusions. At 48 h after N addition, thylakoid membranes were fully developed in most cells. The total area of high electron-dense inclusions decreased.

### Effect of PG Supplementation to N-Starved Synechocystis Cells

**FIGURE 8:**
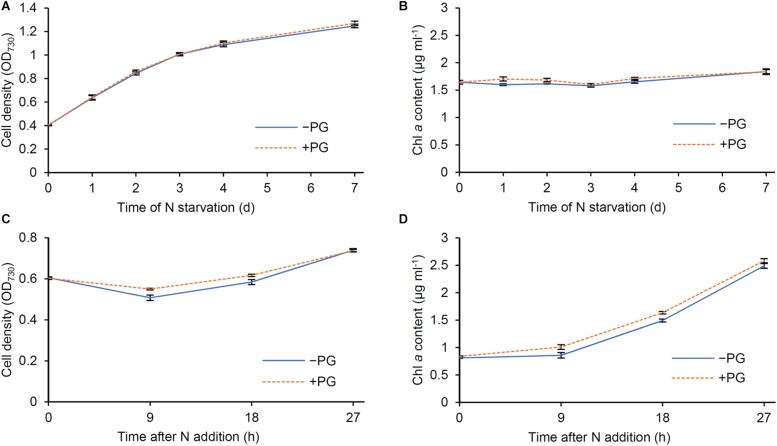
Effect of phosphatidylglycerol (PG) supplementation to cells during N starvation and recovery. Changes of **(A,C)** OD_730_ and **(B,D)** Chl *a* content per milliliter of culture during **(A,B)** N starvation and **(C,D)** recovery were analyzed in the absence (−PG) and presence (+PG) of PG. Data are means ± SE from six independent experiments.

The biosynthesis of PG, which is required for Chl *a* biosynthesis in *Synechocystis* (Kopečná et al., 2015), occurred faster than that of Chl *a* after N addition (Figures 1F, 5B). Because *Synechocystis* increases intracellular PG content by taking up external PG in the growth medium (Hagio et al., 2000), we tested whether external PG affects the Chl *a* metabolism during N starvation and recovery. We grew wild-type *Synechocystis* cells in the presence (+) or absence (−) of 20 μM PG and measured OD_730_ and Chl *a* content during N starvation and recovery. During N starvation, cell density represented by OD_730_ of +PG cells was comparable with that of −PG control (Figure 8A). In both +PG and −PG cells, Chl *a* content in culture was constant (Figure 8B), consistent with the data in Figure 6A. The data show that the presence of PG in growth media does not affect cell growth and Chl *a* metabolism during N starvation. After transfer to the N-sufficient medium, OD_730_ of −PG cells first slightly decreased and then increased (Figure 8C), as observed in Figure 1B. However, this initial decrease in OD_730_ was suppressed in +PG cells. Total Chl *a* content in culture began to increase after 9 h of N addition in −PG cells (Figure 8D). In +PG cells, Chl *a* content was higher than that in −PG cells at 9 and 18 h after N addition, but became similar to that in −PG cells at 27 h.

## Discussion

### Disorganization of the Thylakoid Membrane Components by N Starvation

The rapid decrease in phycocyanin content per culture during N starvation (Supplementary Figure S3) suggests active degradation of PBSs, which would contribute to N recycling from the phycobiliproteins and also reducing the antenna size of PSII. Meanwhile, total protein content per culture was unchanged during N starvation (Figure 6B). Thus, amino acids derived from degraded phycobiliproteins may be reused for the synthesis of other proteins or polypeptides in N-starved cells. Chl *a* content per culture was also constant during N starvation (Figure 6A), although the amounts of Chl *a* and total protein decreased when normalized by OD_730_ (Figures 1D, 2A). The OD_730_ values themselves slightly increased similar to the increase in cell number (Figure 1A; Krasikov et al., 2012). These data suggest that Chl *a* and total protein content in a cell is decreased by dilution due to cell growth under the N-starved condition.

In N-starved cells, the levels of most proteins including RbcL moderately decreased compared with those in N-sufficient cells (Figures 2C,D), consistent with the gradual decrease in total protein content based on OD_730_ (Figure 2A). However, similar to phycobiliproteins, PSII protein levels strongly decreased in N-starved cells (Figure 2D), which was accompanied by a substantial decrease in the oxygen-evolving activity (Figure 3C). Consistent with our data, Ogawa and Sonoike (2016) determined the “true” maximum quantum yield of PSII (Fv/Fm) in *Synechocystis* by eliminating the interference from phycocyanin fluorescence and revealed that the Fv/Fm around 0.8, which was similar to the value observed in land plants, substantially decreased with N starvation. Inactivation of PSII may reduce a risk of photodamage from light energy that exceeds the photosynthetic capacity in N-starved cells with decreased cellular metabolism (Forchhammer and Schwarz, 2019).

In contrast to PSII proteins, PSI reaction center proteins PsaA and PsaB were relatively abundant even under N starvation (Figure 2D), which agrees with the proteomic data that PSI proteins are less degraded than PSII proteins with N starvation (Spät et al., 2018). Consistent with these results, Y(ND), which reflects the donor-side limitation of the PSI photochemistry, was remarkably high in N-starved cells, whereas the acceptor-side limitation represented by Y(NA) was negligible (Figures 4B,C). The data suggest that the linear electron transport from PSII to PSI is strongly downregulated in response to N starvation. In N-starved cells, the selective decrease of PSII components (Figure 2D) and the lowered PSII photochemical efficiency (Ogawa and Sonoike, 2016) would repress the linear electron transport to prevent over-reduction of PSI and consequent production of reactive oxygen species. Reduced thylakoid membranes with inactive PSII and active PSI were also observed in dark-grown *Synechocystis* cells (Barthel et al., 2013), implying an importance of the high PSI and low PSII activity in dormant *Synechocystis* cells. Low Y(NA) in N-starved cells suggests sufficient electron transport from P700 to downstream pathways including the cyclic electron transport system. Doello et al. (2018) revealed that the N-starved chlorotic cells maintain ATP levels to 20–25% of nutrient-sufficient cells. As discussed by Forchhammer and Schwarz (2019), the cyclic electron transport around PSI may be retained in N-starved cells to maintain ATP levels and cell viability during chlorosis.

During N starvation, the amount of membrane lipids also decreased based on OD_730_, but with a smaller extent than those of Chl *a* and total protein content (Figure 5A). In fact, unlike Chl *a* and total protein, the amount of total membrane lipid per culture increased even under the N-starved condition (Figure 6C). Glycerolipids constitute the plasma membrane and the outer membrane besides thylakoids and thus a certain amount of lipids is constantly required to maintain cell integrity. Of note, lipid analysis in the cyanobacterium *Anacystis nidulans* revealed that the cytoplasmic membrane contained more galactolipids (MGDG and DGDG) and less anionic lipids (PG and SQDG) than the thylakoid membrane (Murata et al., 1981; Omata and Murata, 1983). Therefore, biosynthesis of membrane lipids, particularly galactolipids, may be partially maintained in N-starved cells to support their low growth activity. Under the N-starved condition, PG content decreased based on OD_730_, but was unchanged on a culture basis. Thus, dilution due to cell growth may cause the decreased PG content in N-starved cells, as in the case of the Chl *a* and total protein content. Because external PG addition did not affect OD_730_ and Chl *a* content in N-starved cells (Figures 8A,B), the decreased PG content in N-starved cells would be independent of the suppression of cell growth and Chl *a* accumulation during N starvation.

Fatty acid compositions of membrane lipids also changed in response to N status; the proportions of 16:0 and 18:1 increased and those of 18:2 and 18:3 decreased with N starvation (Figure 5C). Gombos et al. (1987) reported that, in the cytoplasmic membrane of *A. nidulans*, the protein-to-lipid ratio decreased with N starvation. In parallel, the proportion of 16:1 decreased and that of 18:0 increased in all 4 glycerolipid classes in the cytoplasmic membrane of N-starved *A. nidulans*. They propose that the changes from shorter unsaturated fatty acids to longer saturated fatty acids in the cytoplasmic membrane would counteract the fluidizing effect due to the decreased protein-to-lipid ratio in the N-starved cells. The decreased polyunsaturation of fatty acids in *Synechocystis* may also contribute to maintaining a proper fluidity in membranes during N starvation, although fatty acid species used for membrane lipids are largely different between *Synechocystis* and *A. nidulans*.

### Reconstruction of Thylakoid Membrane Systems After N Addition

After N addition to the starved cells, the oxygen evolving activity under saturating light rapidly increased (Figure 3C). Y(I) also quickly increased and Y(ND) inversely decreased after N addition (Figures 4B,C). These data suggest that the linear electron transport to PSI was rapidly improved after N addition to increase net photosynthetic activity. The increased amounts of PSII proteins and photosynthetic pigments after N addition would contribute to the improved linear electron transport. Of note, the respiratory activity was transiently enhanced by N addition (Figure 3A). The rapid decrease in Y(ND) under growth light between 0 and 15 h after N addition (Figure 4B), which coincided with the transient increase in the O_2_ consumption rate (Figure 3A), may reflect electron donation from the respiratory flows to plastoquinone pools and the resulting relaxation of the donor-side limitation of P700. Doello et al. (2018) reported that glycogen breakdown is essential for N-starved cells to increase respiration after N addition and resuscitate from chlorotic growth. The transient increase in respiration and ATP concentration in response to N addition would help regenerate PSII and reconstruct the efficient linear photosynthetic electron transport system. However, considering that the change in oxygen evolving activity after N addition preceded the increases in the amounts of PSII proteins and pigments, other mechanisms may also be involved in the improved photosynthetic activity at the early recovery stage, particularly within 9 h after N addition.

In addition to the photosynthetic components, glycerolipid content increased in response to N addition to starved cells (Figures 5A,B). Whereas the increase in glycolipid (MGDG, DGDG and SQDG) content was gradual, that in PG content was rapid particularly between 9 and 24 h after N addition. This observation is consistent with the special importance of PG for the structure and function of photosystems (Kobayashi et al., 2016). Unlike glycolipids that are mostly used to form the lipid bilayer of thylakoid membranes, a large proportion of PG is allocated to photosystem complexes as a structural component (Kobayashi et al., 2017). Biosynthesis of PG, but not those of glycolipids, may be strictly co-regulated with those of other photosystem components. Considering that depletion of PG in the *Synechocystis* mutant lacking PG biosynthesis (*pgsA*) perturbed the structure and activity of PSII (Hagio et al., 2000; Sakurai et al., 2003, 2007; Itoh et al., 2012) and biosynthesis of Chl *a* (Kopečná et al., 2015), cellular PG content would be tightly associated with the PSII functionality and Chl *a* biosynthesis. Of note, addition of PG to PG-depleted *pgsA* mutants quickly recovered some impairment of the PSII activity (Hagio et al., 2000; Sakurai et al., 2003; Itoh et al., 2012). Furthermore, we observed that addition of PG to wild-type *Synechocystis* slightly accelerated Chl *a* accumulation during the recovery from N starvation (Figure 8D). Because the increase of PG content occurred faster than that of Chl *a* content after N addition, the rapid recovery of PG content may contribute to activating the Chl *a* biosynthesis and photosynthetic activity during recovery from N starvation. The OD_730_ kinetics during the recovery process was also affected by addition of PG to wild-type cells (Figure 8C). Although the mechanism for the transient decrease in OD_730_ between 9 and 18 h after N addition is unknown, increased PG content may somehow affect cellular processes that alter light scattering properties of recovering cells.

In N-starved cells, thylakoid convergence zones were rarely observed as compared with N-sufficient cells (Figures 7A,C). However, these zones regenerated after N addition to the starved cells. The analysis of *Synechocystis* mutant that lacks the small membrane-bending protein CurT suggests that the convergence zones formed by CurT function in the assembly and accumulation of PSII, but not PSI (Heinz et al., 2016). Moreover, *in situ* cryo-electron tomography of *Synechocystis* cells revealed that the convergence membranes have many membrane-associated ribosomes and may serve as the translation site (Rast et al., 2019). Thus, disappearance and reappearance of the convergence zones with N starvation and N addition, respectively, are likely to be linked with the changes in the synthetic activity of PSII proteins under these conditions. Although membrane lipids strongly affect the architecture and functions of thylakoid membranes in plant chloroplasts (Hölzl et al., 2009; Kobayashi et al., 2013, 2015; Fujii et al., 2019a), whether specific lipids are involved in the formation and function of convergence zones in *Synechocystis* awaits future studies.

## Data Availability Statement

All datasets generated for this study are included in article/Supplementary Material.

## Author Contributions

YO and KA conceived the research. YO, KK, AY, and MS conducted the experiments. YO, KK, AY, MS, and KA performed data analysis. KK and KA wrote the manuscript.

## Conflict of Interest

The authors declare that the research was conducted in the absence of any commercial or financial relationships that could be construed as a potential conflict of interest.
