# Supplementary material for: Relationship Between Glycerolipids and Photosynthetic Components During Recovery of Thylakoid Membranes From Nitrogen Starvation-Induced Attenuation in Synechocystis sp. PCC 6803
Source: Front Plant Sci. 2020 Apr 15;11:432. doi: 10.3389/fpls.2020.00432 (PMC7175274; doi:10.3389/fpls.2020.00432)
Supplement: Supplementary file 1 [file Data_Sheet_1.PDF]

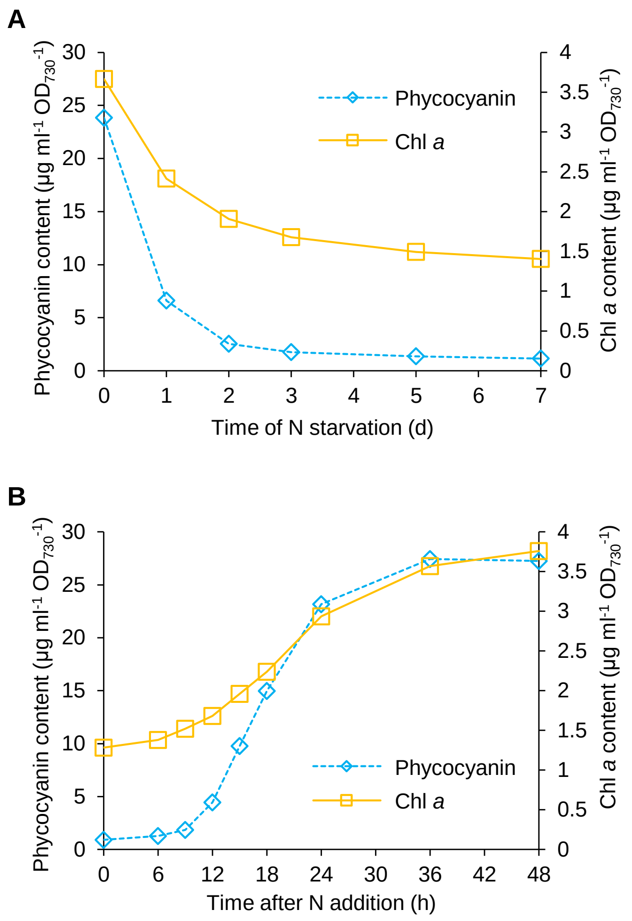


**Supplemental Figure S1.** Estimation of phycocyanin and chlorophyll (Chl) *a* content from absorption spectra of the cells (Figure 1C,E) during **(A)** N starvation and **(B)** recovery. *Synechocystis* cells were grown in the N-free medium for 7 d **(A)** and then were transferred to the N-containing medium and grown for indicated times **(B)**. Data are means from two independent experiments.

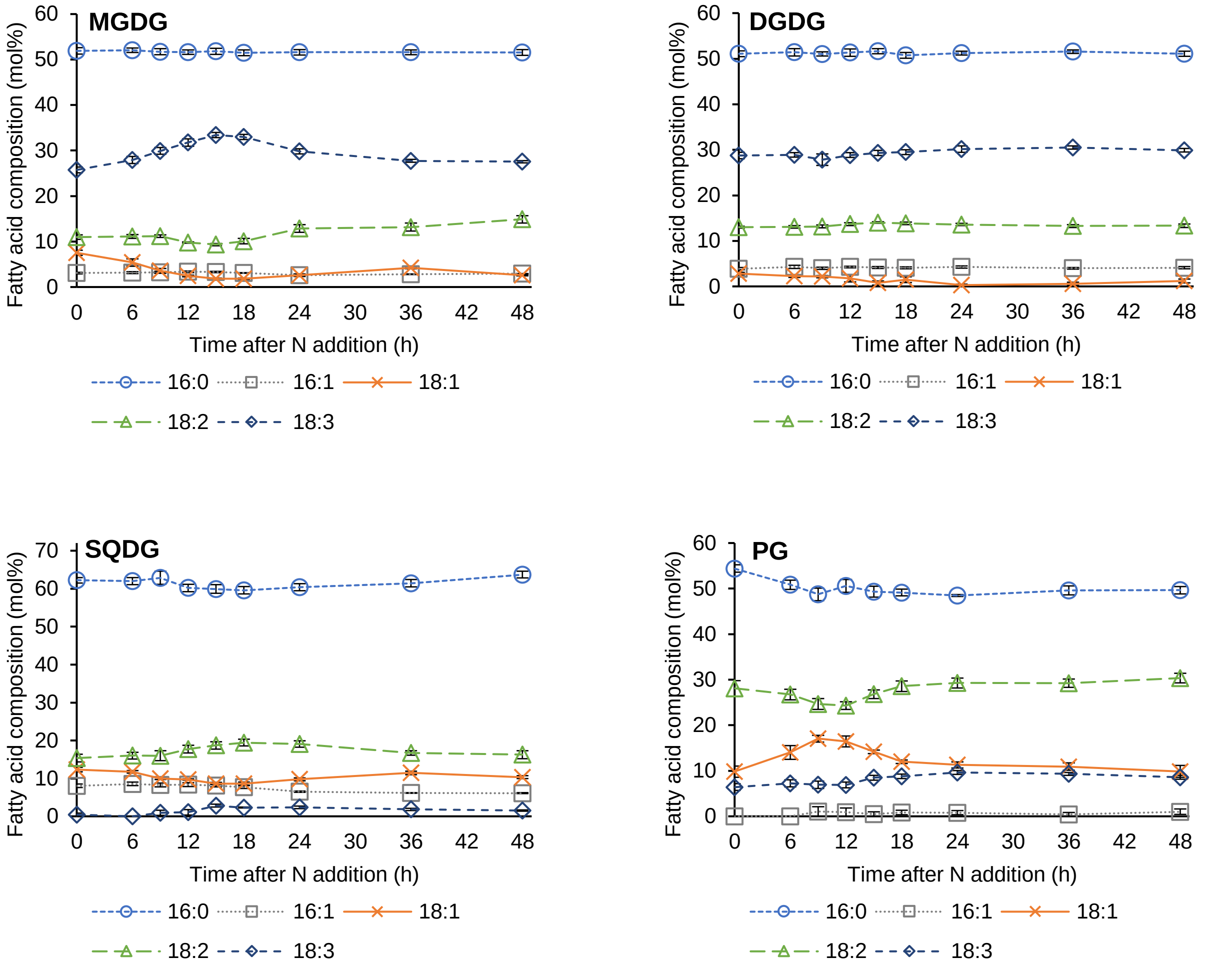


**Supplemental Figure S2.** Changes of fatty acid composition in each glycerolipid during the recovery from N starvation. The 16:2 and 18:0 fatty acids were very minor in all glycerolipid classes and thus were omitted from the data. *Synechocystis* cells were grown under the N-starved condition for 7 d and then grown in the N-containing medium for indicated times. Data are means ± SE from four independent experiments.

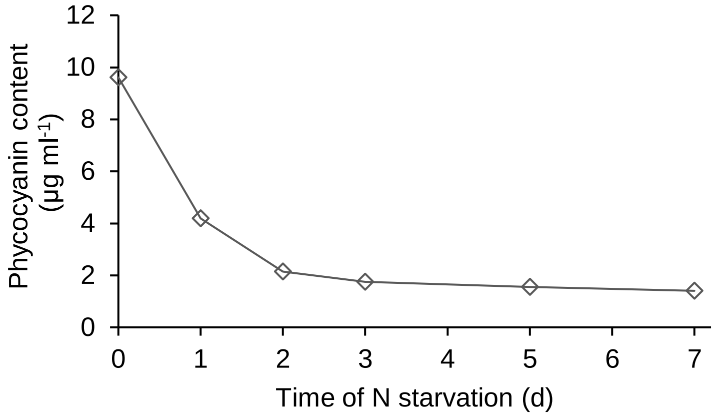


**Supplemental Figure S3.** Changes of phycocyanin content based on culture volume during N starvation. Phycocyanin content based on $OD_{730}$ (Supplemental Figure S1A) was recalculated with the $OD_{730}$ data in Figure 1A to estimate the content per milliliter of cell culture. Data are means from two independent experiments.
